# Supplementary material for: Laser microdissection, proteomics, and multiplex immunohistochemistry: a bumpy ride into the study of paraffin-embedded fetal and pediatric lung tissues
Source: Front Med (Lausanne). 2023 Aug 29;10:1191205. doi: 10.3389/fmed.2023.1191205 (PMC10495683; doi:10.3389/fmed.2023.1191205)
Supplement: Supplementary file 5 [file Data_Sheet_1.DOCX]

**Supplementary data**

**Supplementary table 1** - Descriptive list of samples used in proteomic data analysis.

**Supplementary table 2** - Annotated protein cellular localization (nuclear, extracellular matrix, cytoplasm, and plasma membrane).

**Supplementary table 3 -** Sequential Immunoperoxidase Labeling and Erasing (SIMPLE) procedure for the different antibodies.

**Supplementary table 4** – List of Biological Processes enriched in postnatal and fetal bronchioli, compared to alveoli and canaliculi, respectively.

**Supplementary figure 1 –** (**A**) Principal component analysis (PCA) and (**B**) protein expression levels heatmap of the 4 different LMD compartments collected, postnatal bronchioli (red) and alveoli (orange), fetal bronchioli (blue) and canaliculi (turquoise). Each dot in the PCA graph corresponds to an individual sample and sample numbering is in correspondence to supplementary table 1. In the heatmap, missing values are represented in black. N=5 for each group.

**Supplementary figure 2** – Venn diagrams depicting the annotated protein localizations (nuclear, extracellular matrix, cytoplasm, and plasma membrane) for both postnatal (left) and fetal (right) tissue comparisons.

**Supplementary figure 3 - (A)** Representative images for RAGE (red) and SOX-9 (green) expression in fetal tissues of 19 and 21 gestational weeks (GW) that were used for proteomic analysis. (**B**) Magnification of the region of interest from the upper panel, where SOX-9 is either absent or found at low levels (arrows) in 19 GW fetal samples, while absent at 21 GW. Again, loss of SOX-9 is concomitant with RAGE expression in early alveolar type I cells (in red). SOX-9 can also be found in the cartilage tissue (arrow heads). Hematoxylin is visualized in blue. N=3 replicates for each fetal tissue sample.
