# Supplementary figures and images for: Laser microdissection, proteomics, and multiplex immunohistochemistry: a bumpy ride into the study of paraffin-embedded fetal and pediatric lung tissues
Source: Front Med (Lausanne). 2023 Aug 29;10:1191205. doi: 10.3389/fmed.2023.1191205 (PMC10495683; doi:10.3389/fmed.2023.1191205)

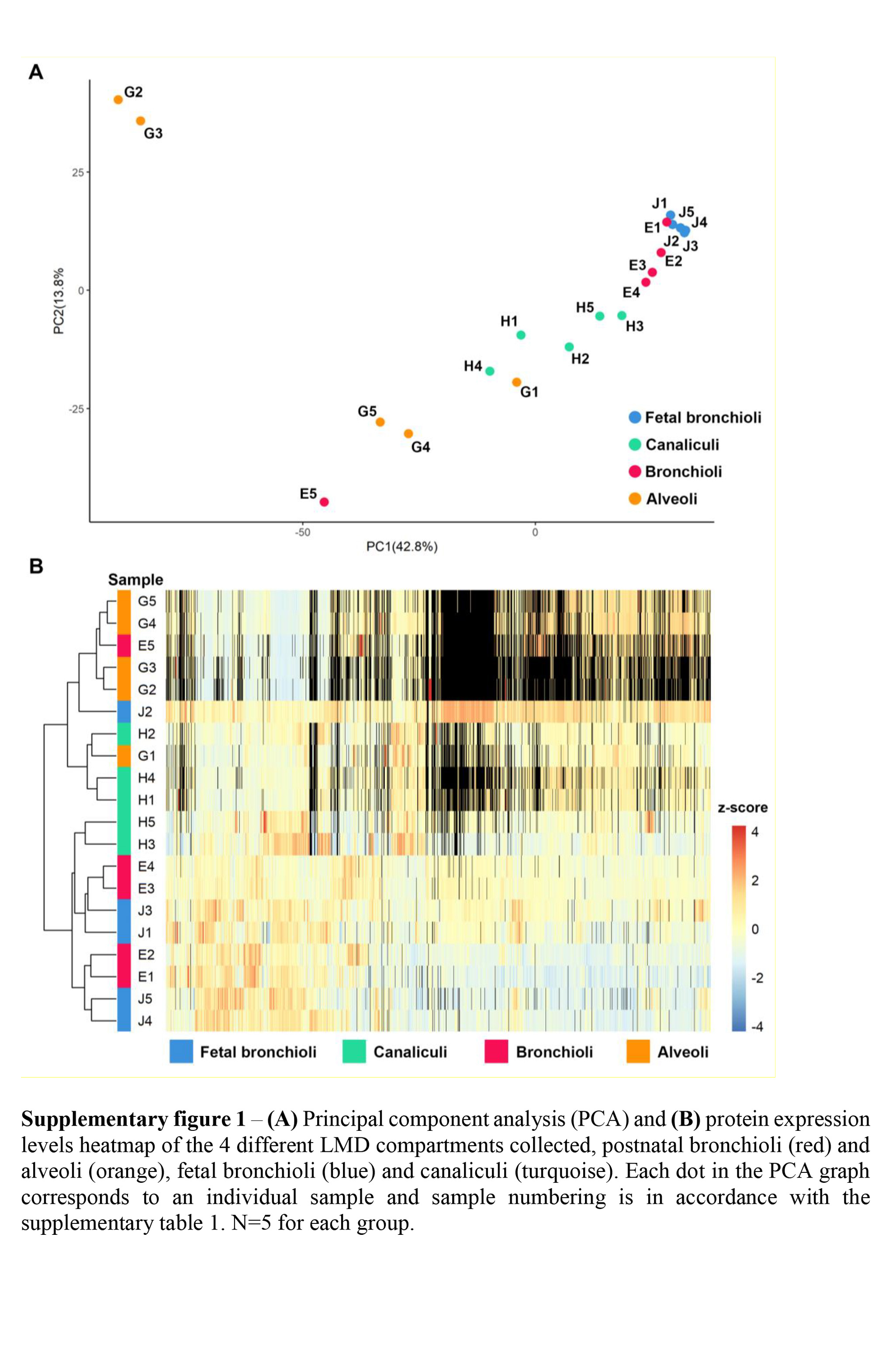

Supplement: Supplementary file 6 [file Image_1.JPEG]
